# Coptisine Suppresses Mast Cell Degranulation and Ovalbumin-Induced Allergic Rhinitis

**DOI:** 10.3390/molecules23113039

**Published:** 2018-11-21

**Authors:** Shuilian Fu, Saihong Ni, Danni Wang, Tie Hong

**Affiliations:** Department of Pharmacology, School of Pharmaceutical Sciences, Jilin University, Changchun 130021, China; fusl16@mails.jlu.edu.cn (S.F.); nish16@mails.jlu.edu.cn (S.N.); wangdn17@mails.jlu.edu.cn (D.W.)

**Keywords:** coptisine, mast cell, allergic rhinitis

## Abstract

Coptisine is one of the main components of isoquinoline alkaloids in the coptidis rhizome. The effect of coptisine on allergic rhinitis has not been investigated. In this study, we report the effects and mechanisms of coptisine using monoclonal anti-2,4,6-dinitrophenyl-immunoglobulin (Ig) E/human serum albumin (DNP-IgE/HSA)-stimulated rat basophilic leukemia cells (RBL-2H3 cells) in vitro and an ovalbumin (OVA)-induced allergic rhinitis (AR) in mice. The results showed that coptisine markedly decreased the levels of β-hexosaminidase, histamine, interleukin (IL)-4, and tumor necrosis factor (TNF)-α. Coptisine also prevented morphological changes, such as restoring an elongated shape, inhibiting granule release on toluidine blue staining, and reorganizing inhibited filamentous actins (F-actin). Additionally, coptisine blocked the phosphorylation of phosphoinositide3-kinase (PI3K)/Akt (as known as protein kinase B(PKB)) in RBL-2H3 cell. Furthermore, the results showed that coptisine suppressed OVA-induced allergic rhinitis symptoms, such as nasal rubbing and OVA-specific IgE, and histamine, IL-4 and TNF-*α* levels in the serum of AR mice. These data suggested that coptisine should have inhibitory effects on the inflammatory responses of mast cells, and may be beneficial for the development of coptisine as a potential anti-allergic drug.

## 1. Introduction

Allergic rhinitis (AR) is one of the most common allergic inflammatory diseases that effects millions of people worldwide; it is relevant to increased levels of allergen-specific IgE, and derives from the sensitization of nasal mucosa with specific allergens including domestic dust mites, pets, and pollens [1,2]. Clinical symptoms such as nasal rubbing, sneezing, nasal congestion, and obstruction seriously affect patient quality of life [3,4].

Mast cells are widely distributed at the mucosal interface, and thus, act as a key role in allergic diseases including allergic rhinitis, urticaria, atopic dermatitis, and asthma [5]. Mast cells are activated by cross-linking immunoglobulin E (IgE) binding to high-affinity IgE receptors (FcεRI) on their surface, which can bind IgE and induce mast cell degranulation following repeated-allergen stimulation [6]. Then, mast cells secrete numbers of bioactive mediators such as histamine, cytokines, and proteases, which relate with the regulation of innate and acquired immune responses [7]. During mast cell degranulation process, FcεRI-dependent signaling pathways such as phosphoinositide3-kinase (PI3K) family members play an vital role in mast cell-induced mediators [8]. PI3K/Akt (as known as protein kinase B(PKB)) pathway is very important for cell auxesis, metabolism, and inflammatory reaction once PI3K conformation was affected, causing the phosphorylation of Ser473 and Thr308 and generation of p-Akt, which could trigger the phosphorylation of many downstream proteins [9].

Rhizoma coptidis, which is called as ”Huang Lian” in China, is an important herbal medicine that contains alkaloids, such as berberine, coptisine, palmatine, jatrorrhizine [10]. As reported, Coptisine exhibited numerous pharmacological activities, such as antibacterial, anti-hyperlipidemic, anticachectic, and antitumor properties [11,12]. Previous studies have demonstrated that coptisine could block activation of mitogen-activated protein kinase (MAPK), PI3K/Akt and nuclear factor kB(NF-kB) pathways in macrophages, and accordingly inhibit LPS-induced inflammatory reaction [13]. Another study found that coptisine suppressed the IL-1β-stimulated inflammation via inhibiting the expression of NF-kB [14]. These findings indicate the strong anti-inflammatory effects of coptisine, but study on the anti-allergic effects of coptisine is still unclear.

Hence, we investigated the anti-allergic action of coptisine on DNP-IgE/HSA-stimulated rat basophilic leukemia cells (RBL-2H3 cells) and ovalbumin (OVA)-induced allergic rhinitis in mice.

## 2. Results

### 2.1. Effect of Coptisine on Cell Viability

**Figure 1:**
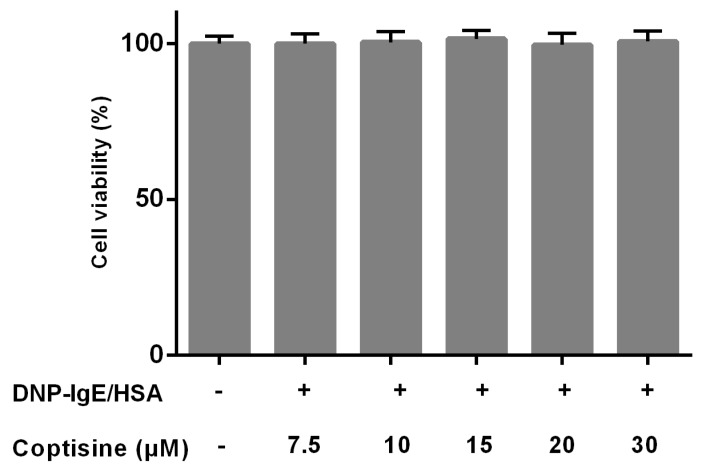
Effect of coptisine on the viability of RBL-2H3 cells. All data were expressed as the mean ± SE.

The effects of coptisine on the cell viability were studied by MTT to ensure that the decreased levels of mast cell granule were not due to the cell death. Various concentrations of coptisine did not obviously affect the cell viability of RBL-2H3 in this experiment (Figure 1).

### 2.2. Effect of Coptisine on the Levels of β-Hexosaminidase and Histamine from Mast Cells

**Figure 2:**
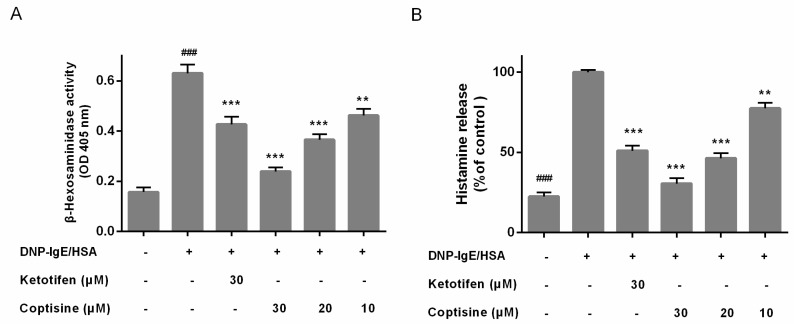
Effect of coptisine on the levels of β-hexosaminidase and histamine from mast cells. Coptisine pretreated (30, 20 or 10 μM) in DNP-IgE/HSA sensitized RBL-2H3 cells. (**A**) The level of β-hexosaminidase; (**B**) The level of histamine (the absolute concentration of histamine for control is 210.64 ng/mL). All data were expressed as the mean ± SE. ** *p* < 0.01, *** *p* < 0.001, in comparison with control group; ### *p* < 0.001 in comparison with control group.

To determine the allergy suppressor effect of coptisine in mast cells, the release of β-hexosaminidase and histamine, two indicators of degranulation, were examined [15]. And Ketotifen fumarate, which used as anti-histamine drug for treating allergic disease by suppressing mast cell activation, was used as the positive control. the release of β-hexosaminidase and histamine were high in the DNP-IgE/HSA group. However, Coptisine and ketotifen fumarate both inhibited the β-hexosaminidase and histamine release of DNP-IgE/HSA-stimulated RBL-2H3 cells (Figure 2A,B).

### 2.3. Effect of Coptisine on IL-4, TNF-α Levels in DNP-IgE/HSA-Stimulated RBL-2H3 Cells

**Figure 3:**
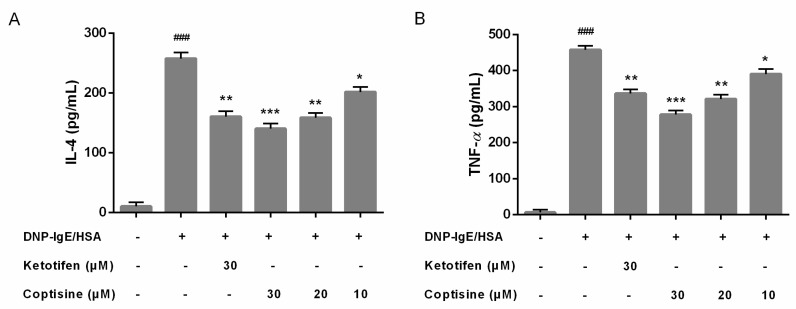
Effect of coptisine on IL-4, TNF-*α* levels in DNP-IgE/HSA-stimulated RBL-2H3 cells. Coptisine pretreated (30, 20 or 10 μM) in DNP-IgE/HSA sensitized RBL-2H3 cells. (**A**) The level of IL-4; (**B**) The level of TNF-*α*. All data were expressed as the mean ± SE. * *p* < 0.05, ** *p* < 0.01, *** *p* < 0.001, in comparison with DNP-IgE/HSA group; ### *p* < 0.001 in comparison with control group.

Mast cell activation could stimulate cytokines release; interleukin (IL)-4 and tumor necrosis factor (TNF)-α are major key proinflammatory cytokines released during mast cell activation [16]. Therefore, we examined the effect of coptisine on the release of IL-4, TNF-α in RBL-2H3 cells. In our present study, pretreatment with coptisine and ketotifen fumarate markedly suppressed the overexpression IL-4 and TNF-*α* (Figure 3A,B).

### 2.4. Effect of Coptisine Granule Release by DNP-IgE/HSA-Stimulated RBL-2H3 Cells

**Figure 4:**
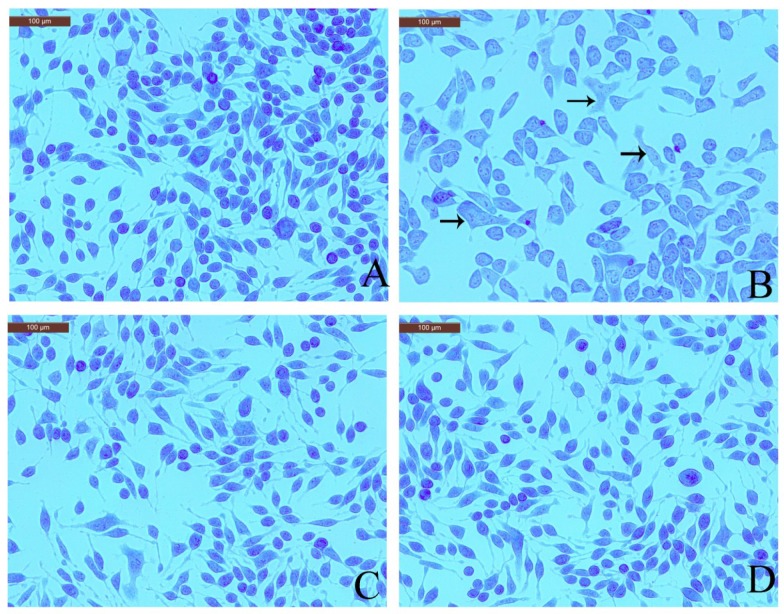
Effects of coptisine with toluidine blue staining in DNP-IgE/HSA-sensitised cells. (**A**) Normal RBL-2H3 cells; (**B**) DNP-IgE/HSA-sensitised RBL-2H3 cells; (**C**) ketotifen fumarate-pretreated RBL-2H3 cells sensitized with DNP-IgE/HSA; (**D**) coptisine (30 μM)-pretreated RBL-2H3 cells sensitized with DNP-IgE/HSA. Arrows in B indicate that the cells morphology became irregular, and purple granules were released outside of the cells.

Toluidine blue staining readily identifies mast cell metachromatic granules against a pale blue background [17]. Therefore, effect of coptisine on toluidine blue staining in RBL-2H3 cells was checked to observe granule release. The normal RBL-2H3 cells were elongated shape and had purple granules stored in the cells. However, the shape of the DNP-IgE/HSA-stimulated RBL-2H3 cells was irregular, and purple granules were released outside of the cell. Pretreatment with coptisine or ketotifen fumarate markedly inhibited the morphological changes and degranulation (Figure 4).

### 2.5. Effect of Coptisine on F-Actin Rearrangement in RBL-2H3 Cells

**Figure 5:**
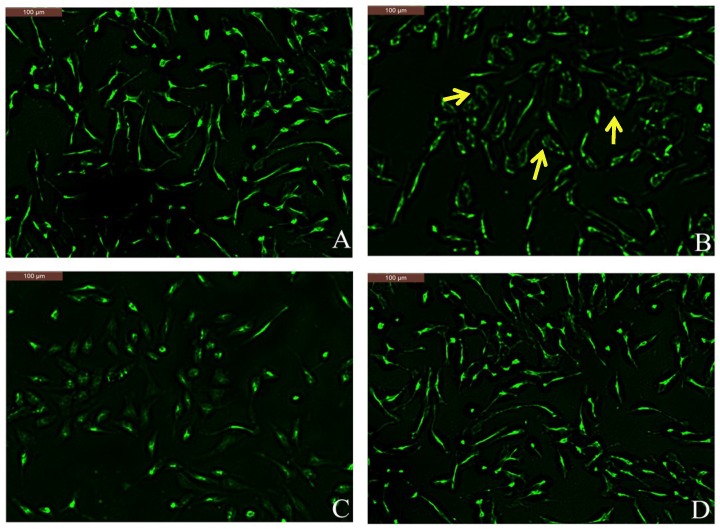
Effects of coptisine on Alexa Fluor-488 phalloidin staining in DNP-IgE/HSA-sensitized cells. (**A**) Normal RBL-2H3 cells; (**B**) DNP-IgE/HSA-sensitized RBL-2H3 cells; (**C**) ketotifen fumarate-pretreated RBL-2H3 cells sensitized with DNP-IgE/HSA; (**D**) coptisine (30 μM)-pretreated RBL-2H3 cells sensitized with DNP-IgE/HSA. Arrows in B indicate that the cells morphology became irregular due to disassembly of the F-actin cytoskeleton.

Actin may play negative regulatory roles in cellular signaling, and its reorganization is required for cell activation events. F-actin is involved in mast cell degranulation and migration [18,19]. Phalloidin specifically combines with F-actin; therefore, we observed F-actin changes in DNP-IgE/HSA-sensitized RBL-2H3 cells after coptisine pretreatment through Alexa Fluor 488-phalloidin staining. The normal RBL-2H3 cells showed spindle shaped, and at the cell periphery F-actin presented uniform distribution (Figure 5A). The shapes of DNP-IgE/HSA-sensitised RBL-2H3 cells become elliptical because of the F-actin cytoskeleton was disassembled (see Figure 5B). Pretreatment with coptisine or ketotifen fumarate inhibited the shape change and the disassembly of the F-actin cytoskeleton (Figure 5C,D).

### 2.6. Effect of Coptisine on PI3K/Akt Signaling in RBL-2H3 Cells

**Figure 6:**
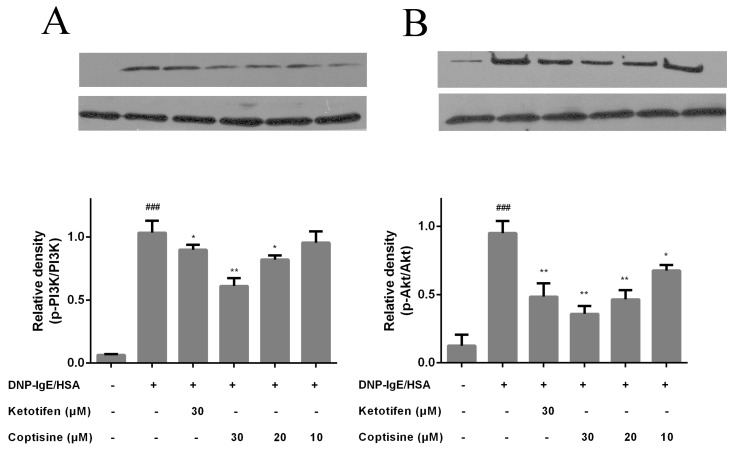
Effect of coptisine on PI3K/Akt signaling in RBL-2H3 cells. The protein level and relative expression of (**A**) PI3K, (**B**) Akt were determined with Western blot. All data were expressed as the mean ± SE. * *p* < 0.05, ** *p* < 0.01, in comparison with DNP-IgE/HSA group; ### *p* < 0.001 in comparison with control group.

PI3K has been implicated in various immune responses and inflammation processes, and mast cell activation is regulated by PI3K/AKT signaling and downstream pathway [20,21]. To investigate the underline mechanism of inhibiting effects of coptisine on mast cell activation, the proteins of PI3K, p-PI3K, Akt, and p-Akt were examined. The phosphorylation of PI3K and Akt were clearly increased in the DNP-IgE/HSA group. By contrast, these proteins were down-regulated by coptisine (Figure 6).

### 2.7. Effect of Coptisine on the Number of Occurrences of Nasal Rubbing in OVA Induced AR Mice

**Figure 7:**
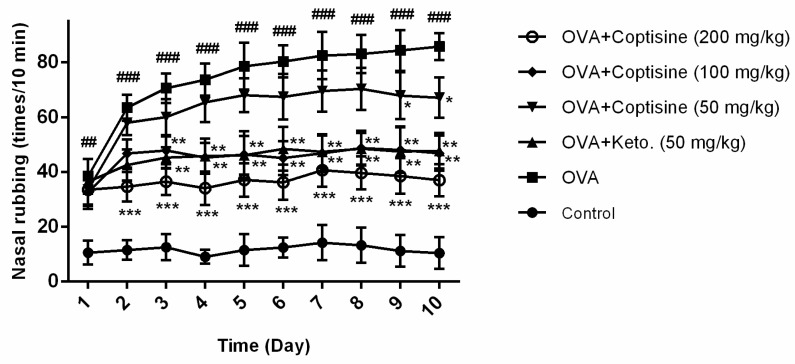
Effect of coptisine on the number of nasal rubbing in OVA induced AR mice. Counting the number of nasal rubbing in the ten min after OVA intranasal injection. All data were expressed as the mean ± SE. ### *p* < 0.001, ## *p* < 0.01 were compared to the control group; * *p* < 0.05, ** *p* < 0.01 and *** *p* < 0.001 were compared to AR mice, n = 10/group.

AR mice showed rapid onset of rubbing after OVA challenge. To examine the effect of coptisine in AR mouse model, coptisine was administrated orally before an in intranasal injection OVA challenge for ten days, then counted the number of nasal rubbing, typical symptoms of AR [22,23]. The level of rubbing was obviously rising in the group receiving OVA compared to the normal mice. By contrast, this effect was suppressed by administering coptisine or ketotifen fumarate (Figure 7).

### 2.8. Effect of Coptisine on OVA-Specific IgE, Histamine Levels in OVA Induced AR Mice

**Figure 8:**
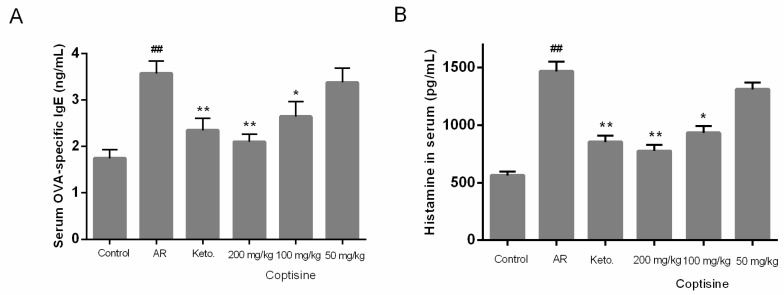
Effect of coptisine on OVA-specific IgE, histamine levels in OVA induced AR mice. The level of OVA-specific IgE (**A**); The level of histamine (**B**) All data were expressed as the mean ± SE. ## *p* < 0.01 were compared to the normal group; * *p* < 0.05 and ** *p* < 0.01 were compared to AR mice, n = 10/group.

To determine the effect of coptisine on allergic response in vivo, the levels of OVA-specific IgE, histamine were measured. Levels of OVA-specific IgE, histamine were markedly increased in AR mice. OVA-specific IgE, histamine levels decreased markedly in the groups receiving coptisine in the AR mice (Figure 8A,B).

### 2.9. Effect of Coptisine on IL-4 and TNF-α Levels in OVA Induced AR Mice

**Figure 9:**
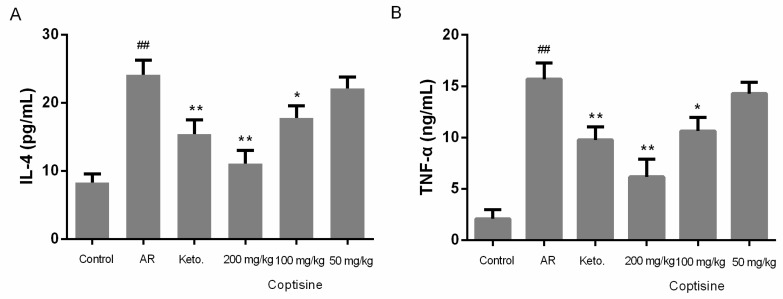
Effect of coptisine on IL-4 and TNF-*α* levels in OVA induced AR mice. Mice were intraperitoneally injected with OVA (50 μg) in aluminum hydroxide (2 mg) once every two days at day 0–14. Then, mice were sensitized by instilling 10 μL of 10% OVA into the bilateral nasal cavities for ten consecutive day. Mice received coptisine or ketotifen fumarate before the intranasal OVA challenge for ten day. The levels of (**A**) IL-4, and (**B**) TNF-*α* were measured by the ELISA method. All data were expressed as the mean ± SE. ## *p* < 0.01 were compared to the control group; * *p* < 0.05, ** *p* < 0.01 were compared to AR mice, n = 10/group.

The allergic response in nasal mucosa challenged with OVA is characterized by common symptoms of AR and the production of proinflammatory cytokines, such as IL-4 and TNF-*α* [2]. To evaluate the regulatory effect of coptisine on the expression of proinflammatory cytokines, we examined the IL-4 and TNF-*α* levels in serum. IL-4 and TNF-*α* levels were clearly increased in AR mice. IL-4 and TNF-*α* decreased markedly in mice receiving coptisine in the AR mice (Figure 9A,B).

## 3. Discussion

Cell-based techniques, such as in vitro approaches, are vital strategies in drug discovery that will save further development costs in early drug screening and discovery [24]. RBL-2H3 cell line is widely used to study IgE-mediated mast cell activation due to strong surface expression of the IgE receptor FcεRI [25,26]. In our study, we used the DNP-IgE/HSA-sensitized RBL-2H3 cell model to study effects of coptisine on mast cell activation.

In IgE-mediated mast cell activation, the cells begin to product cytoplasmic granules with proinflammatory and pro-allergic mediators, which are released into the surrounding environment [27]. Degranulation is a fundamental step in mast cell activation to release inflammatory mediators [28]. The cytoplasmic granules contain β-hexosaminidase, an indicator of degranulation, and histamine, to assess the anti-allergic effect of coptisine [27]. Therefore, we explored the degree of degranulation by measuring β-hexosaminidase and histamine release, two important degranulation markers. The results indicated that coptisine suppressed levels of β-hexosaminidase and histamine induced by DNP-IgE/HSA stimulation. IL-4 is considered to be pivotal in the allergic response, since it induces of isotype switching to IgE, up-regulates adhesion molecules, and promotes eosinophil migration [29]. TNF-*α* could promote inflammation, leukocyte infiltration, and chemotaxis of both neutrophils and T cells, and activated mast cells secrete TNF-*α* that is pivotal in allergic reactions [30,31]. Our data showed that coptisine reduces the IL-4 and TNF-*α* levels. Furthermore, we used toluidine blue staining to confirm that pretreatment with coptisine preserved cell morphology, including the recovery of an elongated shape and less granules released. Membrane ruffling facilitates granules into the surrounding environment. Microfilaments, acting as a barrier which could regulate of cell morphology, are critical in mast cell activation processes [32,33]. Thus, we observed changes in the F-actin cytoskeleton with Alexa Fluor 488-phalloidin staining. The results suggest that coptisine suppresses the rearrangement of the F-actin cytoskeleton. Overall, we deduced that coptisine exerts inhibitory effects on Ag-induced mast cell activation in vitro.

To investigate the underline mechanism of inhibiting effects of coptisine on mast cell activation, PI3K, p-PI3K, Akt, p-Akt were examined. Mast cells are activated by cross-linking of allergen-IgE bound to FcεRI on the cell surface, culminating in degranulation releasing mediators that activated PI3K and phosphorylation PI3K activated Akt. Akt regulates the transcriptional activity of NF-kB, and AP-1, which is vital for TNF-*α* expression [34,35]. Our results showed that the IgE-induced phosphorylation of PI3K and Akt were suppressed by coptisine. These data suggested that coptisine inhibited mast cell activation via PI3K/Akt signaling pathway.

Ovalbumin is widely used to induce AR mice models. After OVA challenge sensitizes the mice, it crosslinks with IgE-FcεRI complexes and leads to the mast cell release of mediators, resulting in symptoms of allergic rhinitis such as nasal rubbing. OVA-induced AR mice show nasal allergic symptoms similar to humans [36]. The same allergen exposure activated the release of inflammatory mediators from IgE-binding mast cells, such as histamine [29]. When OVA sensitization and challenge in mice model was undertaken, the levels of OVA-specific IgE and histamine increased [37]. Histamine is produced by mast cells and basophils as part of certain adverse immune responses; moreover, deregulation of histamine is implicated in allergic reactions in addition to many rare diseases [27]. In contrast, the administration of coptisine relieved nasal rubbing and downregulated OVA-specific IgE and histamine content. This result is consistent with a previous study which confirmed that berberine treatment obviously improved nasal symptom scores, and inhibited the elevation of serum IgE due to its antioxidant and anti-inflammatory effects on AR [38]. In conclusion, this current experimental approach provides evidence on the inhibitory effect of coptisine on IgE-mediated allergic response in vitro and OVA-induced mice allergic rhinitis. This study is beneficial for the development of coptisine as a potential anti-allergic drug.

## 4. Materials and Methods

### 4.1. Cell Culture

The RBL-2H3 cells (ATCC#CRL-2256^TM^) were obtained from the National Infrastructure of Cell Line Resource (Shanghai, China) and were cultured in minimum essential medium (MEM) with 15% fetal bovine serum (FBS), 100 μg/mL streptomycin, 100 U/mL penicillin, 1.5 mg/mL sodium bicarbonate, and 110 μg/mL sodium pyruvate at 37 °C in a humidified incubator with 5% CO_2_.

### 4.2. Animals

Female BALB/c mice (20 ± 2 g) were purchased from the Laboratory Animal Center of Jilin University. The animals were fed standard laboratory chow and water ad libitum, and were adapted under laboratory conditions (12 h light/dark cycle, relative humidity 50–60%, temperature 23 ± 2 °C). All animals in this study were performed in strict according to the National Institute of Health Guide for the Care and Use of Laboratory Animals and approved by the Institutional Animal Care and Use Committee of Jilin University.

### 4.3. Cell Viability Assay

After 12 h seeding in 48-well plate (6 × 10^5^ cells/well, 37 °C), the RBL-2H3 cells were incubated with 100 ng/mL monoclonal anti-2, 4,6-dinitrophenyl-IgE (DNP-IgE, Sigma-Aldrich, ;St. Louis, MI, USA) for another 12 h, and then pretreated with coptisine (purity ≥ 98%, Sigma-Aldrich, St. Louis, MO, USA) for 1 h. The pretreated cells were challenged with 250 ng/mL DNP-HSA (Biorearch, Petaluma, CA, USA) for 12 h at 37 °C. MTT (5 mg/mL) was added to each well, and DMSO was added 4 h later. The absorbance was determined at 490 nm using a spectrophotometer.

### 4.4. β-Hexosaminidase and Histamine Release Assay

The release of β-hexosaminidase and histamine were used as markers of mast cell degranulation. After DNP-IgE/HSA sensitization, the cells were incubated in ice bath for ten min to stop the reaction. For β-hexosaminidase assay, the supernatants were centrifuged (300× *g*, 10 min, 4 °C), and 50 μL supernatant was collected and mixed well with 50 μL substrate (1 mM p-nitrophenyl-*N*-acetyl-β-d-glucosaminide in 0.1 M sodium citrate buffer), and incubated at 37 °C for 1.5 h. OD of reaction solution was measured at 405 nm after adding stop buffer.

For histamine release assay, the supernatants of sensitized cells were centrifuged (10,000× *g*, 10 min, 4 °C); then the supernatants were collected, and the content of histamine was measured according to protocol of enzyme linked immunosorbent assay (ELISA) kits (Elabscience Biotechnology Co., Ltd., Wuhan, China).

### 4.5. Inflammatory Cytokines Assay

After being sensitized with DNP-IgE/HSA, cell culture supernatants were collected to measure levels of IL-4 and TNF-*α* according to protocol of ELISA kits (Elabscience Biotechnology Co., Ltd., Wuhan China).

### 4.6. Toluidine Blue Staining

After challenged by DNP-IgE/HSA, cells were washed with phosphate buffered saline (PBS) and then dipped with 250 μL 4% paraformaldehyde/PBS (30 min, RT). The cells were imaged using Leica inverted microscope (Leica Microsystems, CMS GmbH, Wetzlar, Germany) after 30 min toluidine blue dye staining (0.1% *w*/*v*, pH 2.5).

### 4.7. F-Actin Microfilament Staining 

The sensitized cells were pretreated with coptisine, then washed with PBS and fixed using 4% paraformaldehyde/PBS. The fixed cells were washed with PBS and then pretreated with 0.1% Triton X-100/PBS for five min. After that, PBS wash again and stained with Alexa Fluor 488-phalloidin diluted in 1% BSA (1:1000) for 30 min. Finally, examining F-actin fibers at 490 nm excitation and 520 nm emission filters with Leica DM2500 microscope (Leica Microsystems, CMS GmbH, Wetzlar, Germany).

### 4.8. Western Blot

The protein was extracted from sensitized RBL-2H3 cells, and the concentration determined by the Bicinchoninic Acid (BCA) Protein Assay before loading samples. First, proteins were separated with 10% sodium dodecyl sulfate polyacrylamide gel electrophoresis (SDS-PAGE), then transferred to polyvinylidene fluoride (PVDF) membranes. After in 2 h 5% skim milk blockage, blocked membrane was dipped with anti-PI3K, anti-phospho-PI3K, anti-Akt, anti-phospho-Akt (Bioss, Beijing, China) antibodies (12 h, 4 °C), and then dipped with secondary antibodies (1 h, RT). Enhanced chemiluminescence reagent was used for signal visualization.

### 4.9. OVA-Induced AR in Mice

We built an OVA-induced allergic rhinitis model according to previous studies with modulation [39,40]. Mice were intraperitoneally injected with OVA (50 μg) in aluminum hydroxide (2 mg) once every two days on days 0–14. Then, mice were sensitized by instilling 10 μL of 10% OVA into the bilateral nasal cavities for ten consecutive day. Drug treatment groups were orally administrated coptisine (50, 100 and 200 mg/kg) and ketotifen fumarate (50 mg/kg) (Keto. purity ≥ 98%, Sigma-Aldrich) before intranasal OVA challenge at day 15–24. Normal and AR groups were given deionized water alone. The level of nasal rubbing was determined in the 10 min after OVA intranasal provocation. Twenty-four hours later, the serum was collected, and serum levels of OVA-specific IgE, histamine, IL-4 and TNF-*α* were measured using ELISAs.

### 4.10. Statistical Analyses

Data were analyzed with SPSS and expressed as the mean ± standard error (SE). Statistical significance was performed by one-way analysis of variance (ANOVA), and *p* < 0.05 were considered significant.
